# ERP latency contrasts using Dynamic Time Warping algorithm

**DOI:** 10.1186/1471-2202-14-S1-P434

**Published:** 2013-07-08

**Authors:** A Zoumpoulaki, A Alsufyani, M Filetti, M Brammer, H Bowman

**Affiliations:** 1 School of Computing, University of Kent, Canterbury, Kent, CT2 7NZ, UK; 2 Institute of Psychiatry/ Neuroimaging, Kings College London, London, WC2R 2LS, UK

## 

Latency contrasts are central to Event Related Potential (ERP) research. For example, they are used to determine the order and length of cognitive processes or to evaluate how experimental conditions influence processing time. The most popular methods employed by researchers are peak latency, fractional peak and fractional area. However there are difficulties with these methods, which often include acute sensitivity to noise and window placements [1]. In addition, they require parameter settings that are often difficult to justify. In order to address these issues, we propose Dynamic Time Warping (DTW), an algorithm used for measuring similarity between two sequences, and more precisely the use of the warping path and its relationship to the main diagonal as a measure of latency difference. We tested the performance of DTW by comparing it to 25% - 50% fractional area, peak and 50% fractional peak. In addition to the default DTW we also tested the type IIa step pattern, which constraints the resulting warping path [2]. Data were obtained from a deception detection experiment [3] and ERPs were generated from two channels for one condition. Then the second condition was created in two ways: firstly by offsetting the first condition by 100 time points (0.05 ms), and secondly by offsetting the first condition by an amount sampled from a normal distribution with a mean of 100 time points, while also varying the amplitude. In this way, we simulated latency jitter and amplitude variability between conditions as found in real ERP experiments. Each method was applied to windows placed accordingly to relevant experiments. This was done for each one of the channels (P3a at Fz and P3b at Pz). Then noise was added at the power spectrum of human EEG [4], and the performance of each method was evaluated through permutation tests (100 p-values) for different Signal-to-Noise Ratios (SNR). In order to test the methods independently of response bias, we performed ROC (Receiver Operating Characteristic) analysis, where the false positive rate was obtained by generating the second condition without any latency difference. We also compared DTW's sensitivity to window placement against 25% and 50% fractional area, by selecting a fixed time point as the start of the bounding window and then sequentially adjusting the end of the window and calculating the proportion of windows, for which each of the methods failed to determine the correct latency difference.

Our analysis shows that the basic DTW performs at the same level as the fractional area method, which outperforms peak and fractional peak. The typeIIa DTW performs better than all the other methods for most SNRs. Although there is a slight inflation of false positives for high SNRs, the ROC analysis shows that it does not substantially affect DTW's performance. At the same time, DTW shows significantly less sensitivity to window placement than fractional area. These results indicate that DTW is a promising technique for determining latency differences, being more robust to noise and window placement, without being subject to the same number of assumptions or parameterisation as the other methods evaluated.
